# Enhanced Polarization in Ferroelectric Composites via DIW-Controlled Perovskite Nanosheet Orientation

**DOI:** 10.3390/nano16070432

**Published:** 2026-03-31

**Authors:** Yuxin Han, Zhe Zhu, Hexing Liu

**Affiliations:** 1 State Key Laboratory of Silicate Materials for Architectures, School of Materials Science and Engineering, Wuhan University of Technology, Wuhan 430070, China; 257094@whut.edu.cn; 2 State Key Laboratory of Advanced Technology for Materials Synthesis and Processing, International School of Material Science and Engineering, Wuhan University of Technology, Wuhan 430070, China; 287183@whut.edu.cn

**Keywords:** DIW, nanocomposites, nanosheets, remanent polarization, orientation

## Abstract

PVDF has expanded the application of ferroelectric materials in flexible and wearable electronics due to its flexibility, corrosion resistance, ease of processing, and low cost. However, the polarization of ferroelectric polymers is low, with a bottleneck value of 10 µC cm^−2^. In this study, flexible ferroelectric composite films comprising Ca_2_Nb_3_O_10_ (CNO) nanosheets and PVDF were fabricated via direct ink writing (DIW). By modulating the nozzle-to-substrate height in conjunction with flow-induced shear within the syringe and the application of additional shear force at the nozzle, effective alignment of low-content (2 wt.%) CNO nanosheets dispersed in a highly fluid ink was achieved. The enhanced orientation degree of the CNO nanosheets increased the breakdown strength of the PVDF–CNO composite films to 524 MV/m. Furthermore, the remanent polarization (*P*_r_) was significantly increased by 207% compared to pure PVDF films, reaching a value of 11.6 µC cm^−2^. This study provides a simple and efficient DIW-based strategy for improving filler orientation in composites and demonstrates the substantial enhancement in dielectric and ferroelectric properties achievable through such filler alignment.

## 1. Introduction

Ferroelectric poly(vinylidene fluoride) (PVDF) [1] and its copolymers [2] have significantly expanded the applications of ferroelectric materials in flexible wearable electronics [3], owing to their advantages of flexibility, corrosion resistance, ease of processing, and low cost [4]. PVDF is a semi-crystalline polymer and has five crystal forms: α, β, γ, ε, and δ. Among them, the most common form is the α phase, and the one with the strongest polarity is the β phase. However, due to factors such as crystal structure and molecular chain orientation, the polarization of ferroelectric polymers is low [3]. Despite extensive efforts made to enhance their polarization, such as quasi-morphotropic phase boundaries (MPBs) [5], uni-/biaxial stretching [6], and organic–inorganic composites [7,8], the polarization bottleneck is less than 10 µC cm^−2^ [9]. In conventional organic–inorganic composites, increasing the loading of highly polarized inorganic particles or investigating interfacial effects (e.g., dielectric mismatch ratio [10] and compatibility issues [11,12]) yields only marginal polarization improvements. Furthermore, high inorganic filler loadings compromise flexibility. Therefore, it is of great significance to explore how to increase the content of the β phase and the orientation of the dipoles by adding a small amount of filler simultaneously.

In previous studies, researchers have achieved the orientation of fillers within composite materials through methods such as superspreading [13], freeze-casting [14], layer-by-layer self-assembly (LBL) [15], and 3D printing [16]. The phenomenon where droplets rapidly and completely spread at the interface between a gel and an immiscible liquid is called super spreading [13]. This membrane fabrication method is greatly limited by the size and roughness of the gel base. Freezing casting requires the solution to be directedly frozen under a certain temperature gradient [14], which is subject to significant equipment constraints. LBL involves coating a layer of components on the substrate through techniques such as immersion adsorption and spraying, and then obtaining the desired thickness of the membrane through a cyclic assembly process [15]. This process is rather cumbersome and time-consuming. However, DIW has the advantages of a wide range of material selection, a simple fabrication process, high freedom in shape design, and the ability to be produced over large areas. Zhou [17] et al. achieved a doubling of *P*_r_ by DIW PVDF/M-BTO to fabricate PENG, and the piezoelectric coefficient reached 35 pC/N. Yuan [18] et al., after high-energy ball milling the PVDF and PZT nanoparticles together, used DIW to fabricate the composite material, and the *P*_r_ of this composite material reached 6.26 μC/cm^2^, which was 0.42 μC/cm^2^ higher than that of the composite material not fabricated by DIW, demonstrating the ability of DIW to promote the orientation of β-phase chains and the arrangement of dipoles.

Recently, two-dimensional (2D) inorganic materials, benefiting from their surface electronegativity and high specific surface area [19], have garnered significant attention in flexible piezoelectric applications. Materials like montmorillonite (MTM) [20] and MXene [21] can dramatically increase the polar phase content of PVDF at low loadings (<3 wt.%), and the in-plane orientation of 2D materials within the matrix has proven effective in enhancing the piezoelectric response of composites [22]. Two-dimensional (2D) single-layer Ca_2_Nb_3_O_10_ (CNO) nanosheets have high dielectric constant and surface electronegativity [23,24,25], which can induce the content of β phase in PVDF. Moreover, its dielectric constant is 210, which is much higher than that of other reported nanosheets [26]. The higher polarity of these nanosheets can form an effective electric field within the membrane to enhance the polarization efficiency of the material. At the same time, the CNO nanosheets can induce the dipole orientation of the amorphous region of PVDF, thereby enhancing the polarization of the composite material [27]. Nevertheless, their contribution to ferroelectric polarization is less clear. Direct ink writing (DIW), a proven technique for orienting composite fillers, typically relies on high ink viscosity and specific rheological properties [3]. In the DIW process, alignment is achieved during extrusion via shear forces at the rheological yield point, making DIW a suitable preparation method for composites with high filler loadings [28,29], high aspect ratios [30,31], or large filler sizes [16,32,33]. However, this approach is less applicable to systems utilizing low-content, small-sized fillers.

In this work, we fabricated flexible ferroelectric composite films of Ca_2_Nb_3_O_10_ (CNO) nanosheets and PVDF using DIW. Departing from conventional DIW practices that employ high-viscosity inks and rely on limited rheological shear forces during extrusion to align high-content fillers, we utilized a low-viscosity ink combined with optimized printing parameters. This enabled precise control over the orientation of fillers at a low loading (<2 wt.%). Critically, precise orientation control alone facilitated a breakthrough in ferroelectric polarization (11.6 µC cm^−2^), overcoming the inherent bottleneck. This work advances the theoretical understanding of inducing strong polarization in flexible ferroelectric composites.

## 2. Materials and Methods

Manufacture of Synthesized CNO Nanosheets: CNO perovskite nanosheets were synthesized by liquid-phase exfoliation according to a previous report [34].

Printable Ink Formulation: First, the as-synthesized CNO nanosheets of 0, 5.1, 11.8, and 23.7 mg, corresponding to the mass fraction of 0, 0.5, 1, and 2 wt.% respectively, were dispersed in 5 mL dimethylformamide (DMF) (99.9%, Sigma) and ultrasonically treated for 1 min to evenly disperse nanosheets in DMF solvent. Then, 1.1838, 1.1787, 1.1720, and 1.1601 g polyvinylidene fluoride (PVDF) (Kynar^®^, ARKEMA) powders were respectively added into the mixed solution via magnetic stirring at 55 °C for 10 h to obtain stable and printable inks.

Ink Rheology: The rheological properties of the PVDF and the inks containing 0.5, 1, and 2 weight percent of CNO were analyzed using a rotational rheometer (Kinexus Pro+) equipped with 24.9 mm diameter flat plates and a 1 mm gap. Both storage and loss modulus were measured as a function of shear stress, and viscosity was measured using a logarithmic shear rate ramp ranging from 0.01 to 1000 s^−1^. All rheological tests were conducted at 25 °C after a 1 min preconditioning period.

3D Printing: The CNO/PVDF (0 wt.% to 2 wt.%) inks were transferred into 5 cc dispensing barrels (DYPJ-0014, REGENOVO, China) and stored at 23 °C prior to printing. The printing was carried out through a piston-driven 3D extrusion system (Bio-Architect^®^ SR, REGENOVO, China) through a nozzle with 0.11 mm internal diameter (DYPJ-0022, REGENOVO, China) onto a clean glass substrate. Throughout printing, the barrel and the stage were kept at a temperature of 23 °C. A one-layer film was printed following a raster pattern with 110 μm spacing at 4 mm s^−1^ x–y speed under 4.2 Bar extrusion pressure with total dimensions of 30 mm in width and length. The print difference between shear mode and drag mode is the height of the needle to the substrate. In drag mode, the distance from the needle to the substrate should be greater than the inner diameter of the needle. Conversely, when printing in shear mode, the distance from the needle to the substrate should be less than the inner diameter of the needle. Subsequently, the films were placed in a vacuum oven at 60 °C for 4 h. Finally, the film was quenched at 200 °C for 10 min.

Contact Angle Test: The contact angle of different ink droplets on the glass substrate was measured using a contact angle measuring instrument. The manufacturer of the contact angle measuring instrument is Dataphysics, and the model is OCA35.

Phase Characterization: The phase structure of PVDF was analyzed by the X-ray diffractometer (Rigaku Smartlab) using the model Rigaku Ultima III with a scan rate of 2 degrees per minute. FTIR (Nicolet6700) was used to obtain the absorption spectra of the PVDF and PVDF/CNO composites in the range of 500−4000 cm^−1^.

Crystallinity: The crystallinity of the PVDF and PVDF–CNO composites were obtained using DSC (STA449F3). The samples were placed in a ceramic crucible at 25 °C and heated at 10 °C/min to 200 °C under a nitrogen gas flow (20 mL min^−1^).

Wide-Angle X-ray Scattering (WAXS): WAXS (BrukerNanostar) was used to measure the prepared films by Cu-Kα (λ = 0.154 nm) radiation provided by copper X-ray tube at 50 Kv voltage and 60 mA current. Detector to sample distance was 50 mm.

Microscopic Morphology Characterization: The cross-sectional morphology of the sample was observed using a scanning electron microscope (SEM, JSM 7610F Plus). The film was embedded in resin, and the specimen was prepared via an ultramicrotome (Leica UC7). The orientation of nanosheets in the cross-section was further examined by transmission electron microscopy (TEM, JEM-1400Plus).

Dielectric Characterization: Dielectric spectroscopy measurements were performed in the frequency range from 100 Hz to 1 MHz using LCR (Radiant Agilent E4980A) at 1 V. Electric breakdown strength was evaluated via the dielectric withstand voltage test (Beijing Electro-mechanical Research Institute Supervoltage Technique) at a ramping rate of 200 V/s and a limit current of 2 mA. Each film’s Weibull breakdown strength is fitted with 10 points.

Piezoelectricity: The film was placed in silicone oil and polarized with a DC voltage of 200 MV m^−1^ for 5 min. Then, the direct piezoelectric *d*_33_ coefficient of the DIW film was measured using a quasi-static *d*_33_ piezoelectric meter. The polarization–electric field (P-E) curve of DIW films were evaluated on a ferroelectric analyzer (Radiant Technologies Premier II, PolyK CPE1701). (A normal hysteresis loop test was conducted without PUND.)

## 3. Results and Discussions

### 3.1. Rheological Properties of PVDF–CNO Composite Inks

**Figure 1:**
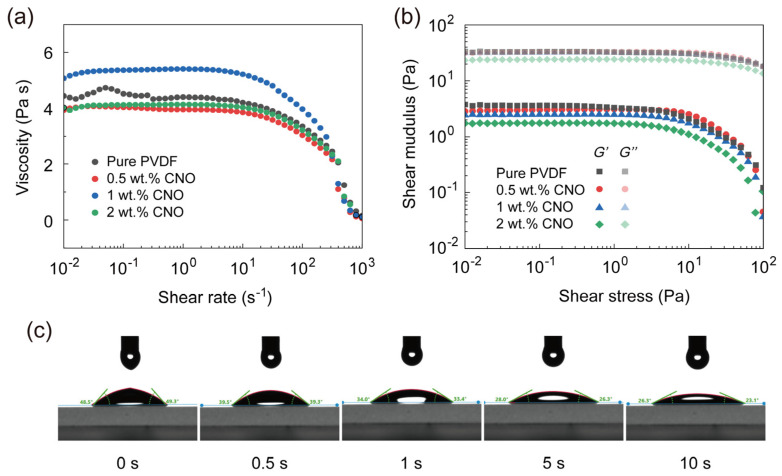
(**a**) Shear thinning and (**b**) shear yielding property of PVDF and PVDF-0.5 wt.% CNO, PVDF-1 wt.% CNO, and PVDF-2 wt.% CNO composite ink. (**c**) PVDF-2 wt.% CNO composite ink spreading process on glass substrate surface.

DIW exhibits broad tolerance towards ink rheology. Printing with inks ranging from low to high viscosity can be achieved by adjusting printing parameters and nozzle size, thereby enabling the realization of the required geometric shapes for devices or ensuring the desired internal material structure. Leveraging this characteristic, and distinct from other approaches utilizing high-viscosity inks to orient high filler loadings [28,35,36,37,38], we formulated low-viscosity inks based on PVDF and PVDF–CNO in DMF. These inks, paired with a small-diameter nozzle (110 µm), were employed to achieve alignment of fillers at low loadings (<2 wt.%). As shown in Figure 1a, the viscosities of all inks were below 10 Pa s, with minimal variation observed across different CNO contents. Crucially, the inks retained their shear-thinning behavior. This implies that during the DIW process, the extrusion of ink through the nozzle subjects it to shear stress, generating velocity gradients within the flow profile which facilitate the alignment of the incorporated fillers. A comparison of the storage modulus (*G*′) and loss modulus (*G*″) for both PVDF and PVDF–CNO composite inks as a function of shear stress is presented in Figure 1b. *G*′ consistently remained lower than *G*′′ across the measured range, confirming that the inks maintained favorable fluidity which is suitable for extrusion through fine nozzles. The contact angle of the PVDF-2 wt.% CNO ink on a glass substrate was also determined. As depicted in Figure 1c, the ink demonstrated favorable wettability, with the contact angle decreasing from approximately 49° to around 25° over time. This behavior aligns with the ink’s good fluidity and is beneficial for maintaining surface flatness during thin-film fabrication.

### 3.2. DIW PVDF–CNO Nanocomposites and the Obvious Orientation Optimization

**Figure 2:**
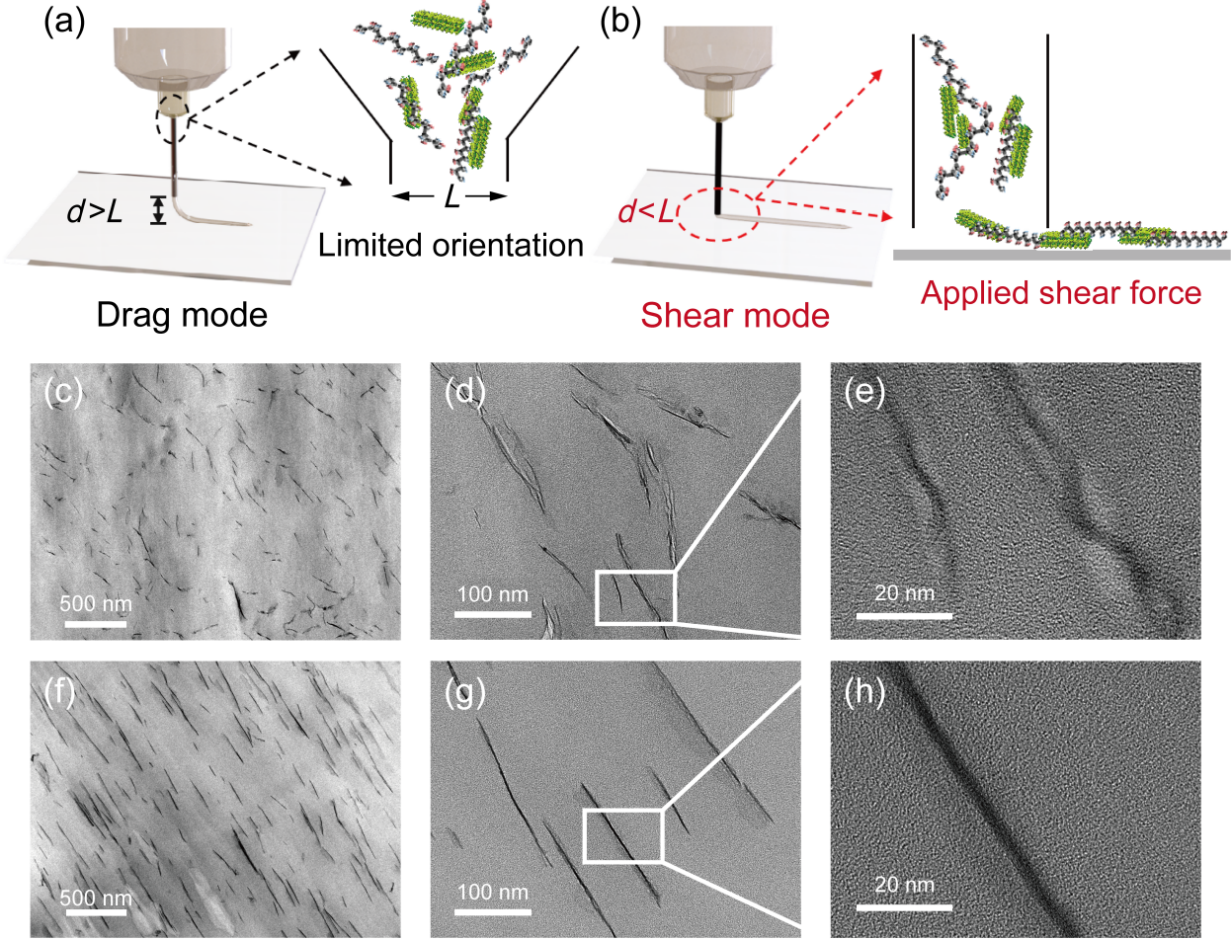
DIW printing process in (**a**) drag mode and (**b**) shear mode. TEM cross-sectional image of 2 wt.% CNO/PVDF nanocomposite films: (**c**–**e**) drag mode, (**f**–**h**) shear mode printing. The scale bars are 500 nm, 100 nm, and 20 nm.

The surface roughness of the pristine and composite films was compared using white-light interferometry. Results from Figure S3a,b reveal that the surface roughness of the DIW-printed pure PVDF film was significantly higher than that of the PVDF-2 wt.% CNO composite film, with measured arithmetic average roughness (*R*a) values of 362 nm and 22 nm, respectively. Meanwhile, we also used AFM to characterize the surface morphology of the 2 wt.% CNO–PVDF composite film, as shown in Figure S6. The obtained *R*a was 64 nm, which was slightly larger than the result of white light interference. However, compared with the pure PVDF film *R*a obtained by white light interference, it was still reduced by 82.3%. To achieve more precise control over the alignment capability of direct ink writing (DIW) for nanosheets at low filler loadings (<2 wt.%), we leveraged the excellent fluidity of the utilized ink. By adjusting the nozzle-to-substrate distance (*d*), two distinct printing modes were established: the drag mode and the shear mode. Figure 2a illustrates the conventional dragging mode, where the d exceeds the nozzle’s inner diameter (*L*). During printing, the extruded ink filament is “dragged” onto the substrate without breakage. However, this mode exhibits limited effectiveness in aligning nanosheets at low filler concentrations. As shown in Figure 2c,d, while the nanosheets appear broadly oriented macroscopically, their distribution is locally disordered, exhibiting significant twisting, crumpling, and multi-layer stacking. Figure 2e clearly depicts stacks of two to three crumpled nanosheets, indicating ineffective dispersion. Nevertheless, when a beam of light passes through a colloid or medium containing tiny suspended particles, a bright “path” can be seen in the medium when observed from a direction perpendicular to the incident light. This phenomenon is known as the Tyndall effect [39]. The pronounced Tyndall effect observed in Figure S1 confirms that the CNO nanosheets possess excellent colloidal stability in DMF due to their negatively charged surfaces, which remains unchanged over time. Conversely, the shearing mode operates with the nozzle-to-substrate distance set smaller than the nozzle’s inner diameter (*d* < *L*), while still permitting continuous ink extrusion (Figure 2b). In this configuration, the nanosheets experience shear forces from two primary sources: (1) the inherent shear flow profile within the nozzle arising from the ink’s rheology, and (2) an additional, significant doctor-blade-like shear force imposed at the nozzle exit due to the confined gap. This dual shear mechanism effectively aligns the low-concentration nanosheets. Cross-sectional transmission electron microscopy (TEM) images of the composite film containing 2 wt.% CNO provide direct and compelling evidence. Figure 2f,j reveal exceptionally high levels of nanosheet alignment achieved under the shearing mode. Progressive magnification (Figure 2g,h) further demonstrates that this dual shear action effectively flattens crumpled nanosheets, promoting monolayer dispersion. The theoretical thickness of monolayer CNO nanosheets has been determined as 1.45 nm [40]. Figure 2h shows a nanosheet thickness of approximately 2 nm, consistent with a monolayer structure. The darker contrast represents the edge-on section of the nanosheet, while the lighter surrounding area corresponds to the projection of its planar domain. This contrast variation likely arises from a slight tilt angle between the cross-section plane and the film surface during TEM sample preparation. We further performed SEM analysis on films printed under shear mode. As shown in Figure S2, both the pure membrane and composite membranes exhibit a thickness of approximately 12 µm. All membranes display a dense morphology devoid of pores or voids. With increasing CNO nanosheet content, the fracture surfaces of the composite membranes reveal a greater number of nanosheet pull-out traces. However, the orientation state of the nanosheets could not be determined from the cross-sectional SEM images.

### 3.3. Interfacial Piezoelectric Polarization Locking

**Figure 3:**
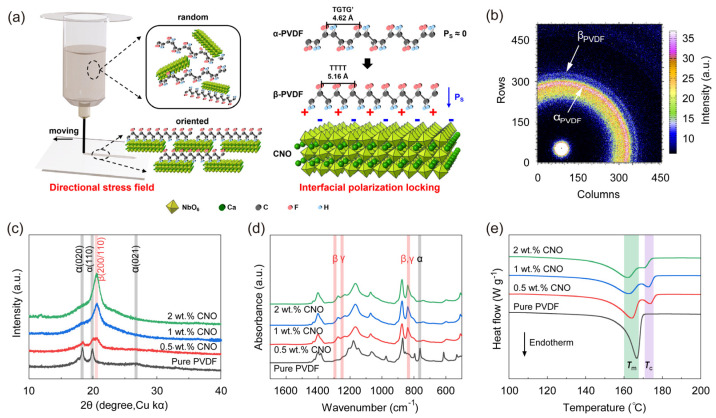
(**a**) Schematic illustration of the polar orientation locking mechanism and characterization of oriented CNO/PVDF composite films: (**b**) 2D WAXS pattern of nanocomposite film with the diffraction rings from β-phase PVDF (β_PVDF_) and α-phase PVDF (α_PVDF_), (**c**) XRD spectra, (**d**) FTIR spectra, (**e**) DSC thermograms of composite films with different CNO content printed under shear mode.

Figure 3a presents a schematic illustration of the shear-mode printing process in the left panel, while the right panel depicts the electrostatic interactions between PVDF molecular chains and CNO nanosheets. CNO nanosheets are composed of corner-sharing NbO_6_ octahedra with Ca atoms situated at the centers of four octahedra. The non-overlapping polar centers result in a negatively charged surface on the CNO nanosheets. PVDF is widely recognized as a typical semi-crystalline polymer, predominantly crystallizing in the α-phase, which represents the thermally stable polymorph at room temperature. In this phase, PVDF chains adopt a trans-gauche-trans-gauche′ (TGTG′) conformation (T: trans, G: gauche+, G′: gauche−), exhibiting antiparallel alignment of molecular dipoles within a non-polar crystal structure. However, upon incorporation of CNO nanosheets, the PVDF chains undergo a conformational transition to the all-trans conformation (*T*_m_ ≥ 4) driven by electrostatic attraction. This conformation is subsequently pinned by local polarization induced from the aligned CNO nanosheets, ultimately facilitating crystallization into the β-phase.

**Figure 4:**
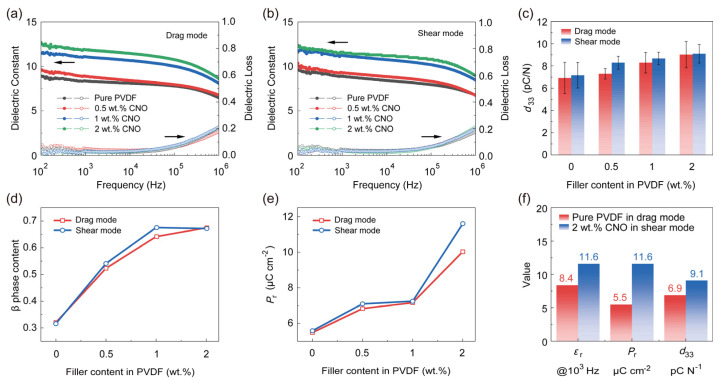
Comparative analysis of (**a**,**b**) *ε*_r_, (**c**) *d*_33_, (**d**) β-phase content, and (**e**) *P*_r_ in composite films fabricated by drag mode versus shear mode. (**f**) Comparison of electrical properties between aligned nanocomposite film and PVDF polymer: *ε*_r_ @ 10^3^ Hz, *P*_r_, and *d*_33_ values.

WAXS (Figure 3b) was employed to probe the crystalline structure of PVDF within the composites. Distinct diffraction features corresponding to the non-polar α-phase and the polar β-phase of PVDF were observed. However, due to the low loading level of the nanosheets, the characteristic scattering ring of CNO was absent in this pattern, as the interlayer spacing fell beyond the detection range of the technique. The phase distribution in the DIW composite films was further analyzed using X-ray diffraction (XRD) (Figure 3c). Characteristic peaks of the PVDF α-phase were observed at 2*θ* = 18.4°, 19.7°, and 26.7°, corresponding to the (020), (110), and (021) crystallographic planes, respectively. For the β-phase, the primary diffraction peak, attributed to the (110/200) planes, was located at 2*θ* = 20.6°, where it overlaps with diffraction peaks from the α-phase. Regarding the γ-phase, characteristic peaks at 2*θ* = 19.2° and 20.04°, assigned to the (020) and (110/101) planes, were also superimposed on the α-phase diffraction features in the 20° region. Owing to the low concentration of CNO nanosheets, no discernible peaks associated with the additive were detected in the XRD patterns. A significant phase transition from the α-phase to the β-phase of PVDF was evident with increasing CNO content. Prior to CNO addition, only peaks corresponding to the α-phase were detected. In contrast, the PVDF-2 wt.% CNO composite film exhibited no discernible α-phase peaks and was predominantly characterized by the β-phase peak at 2*θ* = 20.6°. FTIR spectroscopy was employed to quantify the phase fractions within DIW-processed CNO/PVDF composite films, specifically at CNO nanosheet loadings of 0 wt.%, 0.5 wt.%, 1 wt.%, and 2 wt.% (Figure 3d). The characteristic absorption peak at 766 cm^−1^, assigned to the α-phase, is discernible in the pristine PVDF film but absent in the nanocomposite spectra. This observation indicates a significant suppression of the α-phase upon CNO incorporation. Conversely, the peak at 840 cm^−1^, corresponding to the β phase, emerges exclusively in the CNO/PVDF composites. The β phase fraction (*F*_β_) was calculated using FTIR data by Equation (1) [41]:

(1) Fβ%=IβKβKαIα+Iβ,

Here, *I*_β_ is the intensity of β peak, *I_α_* is the intensity of *α* peak, *K_α_* and *K*_β_ are the absorption coefficients for the peaks at 766 cm^−1^ and 840 cm^−1^, with values of 6.1 × 10^4^ cm^2^ mol^−1^ and 7.7 × 10^4^ cm^2^ mol^−1^, respectively [8]. The calculated *F*_β_ for the CNO/PVDF film containing 2 wt.% CNO nanosheets reached 67.5% (Figure 3d). This value is substantially higher than the *F*_β_ of 35.5% determined for the pristine PVDF film, representing the addition of CNO which significantly increased the content of the polar β phase. DSC thermogram (Figure 3e) showed two endothermic peaks for all composite samples, centered at approximately 165 °C and 172 °C. The peak at 172 °C corresponded to the ferroelectric to paraelectric transition (Curie temperature, *T*_c_), whereby the samples exhibit piezoelectric properties below the *T*_c_ and lose polarization above the *T*_c_. The primary peak at 165 °C corresponds to the melting of the polymer (*T*_m_), with the enthalpy (*∆H*_m_) correlating to the crystallinity (*χ*_c_) following Equation (2) [41]:

(2) $$\chi_{c}=\frac{\Delta H_{m}}{{\Delta H}_{0}},$$

where the enthalpy of melting for completely crystalline PVDF (*∆H*_0_) is given as 104.5 J g^−1^. The crystallinity of pristine PVDF film was determined to be 48.12%. Upon incorporating 0.5 wt.% CNO nanosheets, the crystallinity decreased to 45.72%. Further increasing the CNO loading to 1 wt.% resulted in a continued reduction in crystallinity to 44.01%. However, when the CNO content was elevated to 2 wt.%, a slight increase in crystallinity to 46.77% was observed. This initial decrease is attributed to the progressive restriction of polymer chain mobility imposed by the CNO nanosheets, which hinders PVDF crystallization and reduces crystal thickness. Consequently, the melting temperature (*T*_m_) peak shifts to a lower temperature [42], decreasing from 166.6 °C for the pristine film to 161.7 °C for the composite with 2 wt.% CNO, as documented in Table S1. Conversely, the subsequent modest recovery in crystallinity at the highest filler loading (2 wt.%) is ascribed to the enhanced polarization pinning effect induced by strong electrostatic interactions. This effect promotes the formation of the electroactive phase (as shown in Figure 4d), thereby counteracting the initial crystallinity suppression.

### 3.4. Electric Properties of DIW PVDF–CNO Nanocomposites

To investigate the influence of nanosheet alignment achieved via DIW on the electrical properties of the films, we characterized two distinct printing modes. As depicted in Figure 4a,b, at 1000 Hz, the *ε*_r_ of films fabricated by the drag printing mode increased from 7.1 for the pure PVDF film to 8.9 for the composite with 0.5 wt.% CNO, 11.0 for 1 wt.% CNO, and finally 11.9 for the composite with 2 wt.% CNO. Conversely, films prepared by the shear printing mode exhibited *ε*_r_ increasing from 8.9 (pure PVDF) to 9.3 (0.5 wt.% CNO), 11.2 (1 wt.% CNO), and 11.6 (2 wt.% CNO). The *ε*_r_ of the composites before and after nanosheet alignment showed negligible variation, with both increasing progressively with higher CNO nanosheet loading. This demonstrates that CNO nanosheet orientation has no significant impact on the *ε*_r_ of PVDF–CNO composite films at low filler concentrations. Simultaneously, we compared the effect of nanosheet alignment on the breakdown strength. The Eb was calculated from Weibull statistics and is shown in Figure S5.

(3) $$P\left(E\right)=1-exp\left(-{\left(\frac{E}{E_{b}}\right)}^{\beta }\right),$$

where P(E), *E_b_*, and β are the cumulative probability of the electric failure, the characteristic breakdown strength corresponding to 63.2% probability of failure, and shape parameter, respectively [43]. The *E_b_* of the films printed in the shear mode is always higher than that of the membrane prepared in the drag mode. Furthermore, the breakdown strength of all films followed a consistent trend with increasing CNO loading: an initial decrease, followed by an increase, and a subsequent final decrease. For the composite films, the breakdown strength was consistently higher for those with shear-aligned nanosheets compared to their unaligned (drag-printed) counterparts. The maximum breakdown strength of 524 MV/m was achieved for the aligned composite at 1 wt.% CNO loading, exceeding the 481 MV/m value of the unaligned composite prepared by drag printing. However, when the loading capacity of CNO was increased to 2 wt.%, the breakdown strength of the membranes prepared by the shear mode and the drag mode decreased to 391 MV/m and 356 MV/m, respectively. This demonstrates that fabricating PVDF–CNO composites via the shear printing mode can effectively enhance the breakdown strength of DIW films. This enhancement is attributed to the uniformly dispersed and aligned nanosheet network, which promotes a more homogeneous electric field distribution, mitigating localized field concentrations that cause premature breakdown or leakage. Additionally, nanosheets aligned parallel to the PVDF matrix can effectively intercept the propagation of electrical trees and prolong the breakdown path [44].

**Figure 5:**
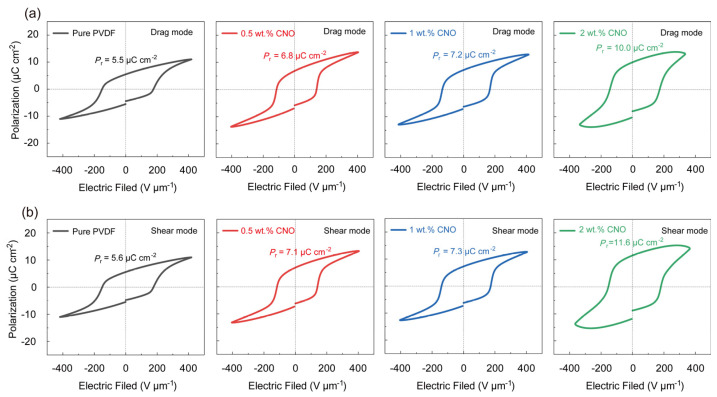
Polarization–electric field hysteresis loop of the films printed in (**a**) drag mode and (**b**) shear mode measured at 10 Hz.

Furthermore, polarization–electric field (P-E) hysteresis loops were acquired for both types of films. (Figure 4e and Figure 5). To ensure that the domains of PVDF undergo sufficient flipping, the P-E hysteresis loops were measured at a frequency of 10 Hz [7,45,46]. The remnant polarization (*P*_r_) of the films increased with higher CNO nanosheet loading. The traces of nanosheet detachment shown in Figure S2 become more numerous as the loading amount increases, which can prove the increase in the content of nanosheets in Figure 5. Notably, the aligned composite films consistently exhibited higher *P*_r_ than their unaligned counterparts. The maximum *P*_r_ reached 11.6 µC cm^−2^ for the aligned film with 2 wt.% CNO, representing 207% of the remnant polarization of the pure PVDF film and is 1.6 µC cm^−2^ higher than that of the non-oriented 2 wt.% CNO–PVDF composite. This significant enhancement in *P*_r_ is attributed to the high dielectric constant of the nanosheets, which helps screen the electrostatic interactions between adjacent polarized regions, reducing the depolarization field [22,47]. Consequently, the dipoles within the PVDF chains can more readily switch under the applied electric field and maintain their switched state, leading to a substantial increase in remnant polarization.

Direct measurements of the longitudinal piezoelectric coefficient (*d*_33_) in poled membranes are presented in Figure 4c. The *d*_33_ increases with higher CNO nanosheet content. While the oriented membranes show slightly higher *d*_33_ than their non-oriented equivalents, the measured difference between the two is relatively small. A comparison of the initial properties of the pristine membrane with the optimized performance is shown in Figure 4f. The oriented 2 wt.% CNO–PVDF composite membrane demonstrates significant improvements: its dielectric constant increases to 11.6, its *P*_r_ reaches 11.6 µC cm^−2^, and its *d*_33_ value rises to ~9.1 pC ^−1^. Combining the advantage of DIW’s customizable patterned preparation, the CNO–PVDF composite film has demonstrated promising application prospects in the field of wearable flexible piezoelectricity.

## 4. Conclusions

In summary, the synergistic effect of shear forces generated by adjusting the ink flow velocity within the syringe and the needle-to-substrate height, along with the application of additional shear stress at the needle tip, effectively aligns low-content (2 wt.%) nanosheets within highly fluid inks. By enhancing the orientation degree of CNO nanosheets, the breakdown strength of the PVDF–CNO composite film was increased to 524 MV/m. Furthermore, the *P*_r_ exhibited a remarkable 207% enhancement compared to the pristine film, reaching 11.6 µC cm^−2^. In addition, the dielectric constant of the composite material remains basically unchanged before and after orientation while the *P*_r_ of the PVDF-2 wt.% CNO composite film increased by 16% compared with that before orientation. This proves that the orientation of CNO nanosheets helps to increase the *P*_r_ of the PVDF–CNO composite materials. This work presents a straightforward and efficient strategy for improving filler alignment in composites fabricated by DIW. It concurrently demonstrates the significant performance gains in dielectric and ferroelectric properties achievable solely through improved orientation.

## Data Availability

Data are contained within the article.

## Supplementary Materials

The following supporting information can be downloaded at: https://www.mdpi.com/article/10.3390/nano16070432/s1, Figure S1: (a–g) CNO was stably dispersed in DMF for 0 h, 2 h, 4 h, 6 h, 18 h, 30 h and 100 h; Figure S2: SEM cross section of (a–c) PVDF, (d–f) 1 wt.% and (g,h) 2 wt.% CNO/PVDF films printed under shear mode; Figure S3: Surface Morphology Characterization of (a) PVDF and (b) 2 wt.% CNO/PVDF Composite Films: White Light Interferometry; Figure S4: Structural characterization of unoriented films fabricated by drag mode: (a) XRD and (b) FTIR Spectroscopy Spectra; Figure S5: Comparative Analysis of Weibull Breakdown Strength for Oriented versus Unoriented Films; Figure S6: The AFM image of the 2 wt.% CNO–PVDF film. The scanning area is 20 μm × 20 μm; Table S1: Enthalpy (*ΔH*_m_), Crystallinity (*χ*_c_), Melting Point (*T*_m_) and Curie temperature (*T*_c_) of DIW PVDF and PVDF—0.5 wt.%, 1 wt.% and 2 wt.% CNO films under shear mode.
