# Supplementary material for: Enhanced Polarization in Ferroelectric Composites via DIW-Controlled Perovskite Nanosheet Orientation
Source: Nanomaterials (Basel). 2026 Mar 31;16(7):432. doi: 10.3390/nano16070432 (PMC13074841; doi:10.3390/nano16070432)
Supplement: Supplementary file 1 [file nanomaterials-16-00432-s001.zip › nanomaterials-4213378-supplementary.pdf]

Supplementary Material

Enhanced Polarization in Ferroelectric Composites via DIW-Controlled Perovskite Nanosheet Orientation

Yuxin Han [1], Zhe Zhu [2] and Hexing Liu [1,*]

[1] State Key Laboratory of Silicate Materials for Architectures, School of Materials Science and Engineering, Wuhan University of Technology, Wuhan 430070, China;
[2] State Key Laboratory of Advanced Technology for Materials Synthesis and Processing, International School of Material Science and Engineering, Wuhan University of Technology, Wuhan 430070, China;
* Correspondence: liuhx@whut.edu.cn

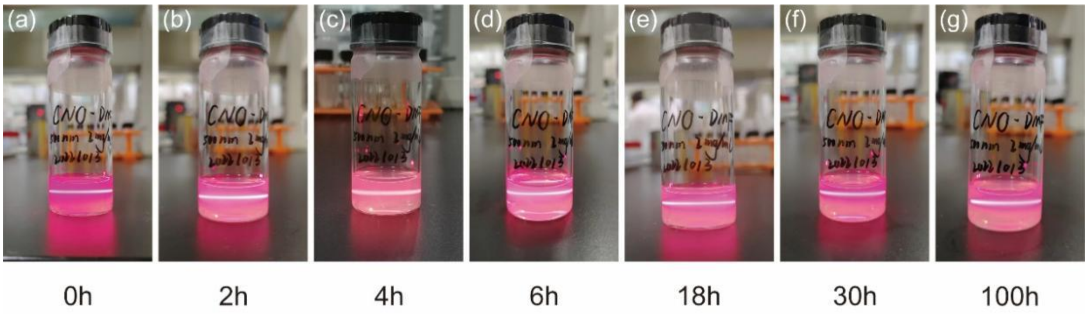

Figure S1. (a-g) CNO was stably dispersed in DMF for 0h, 2h, 4h, 6h, 18h, 30h and 100h.

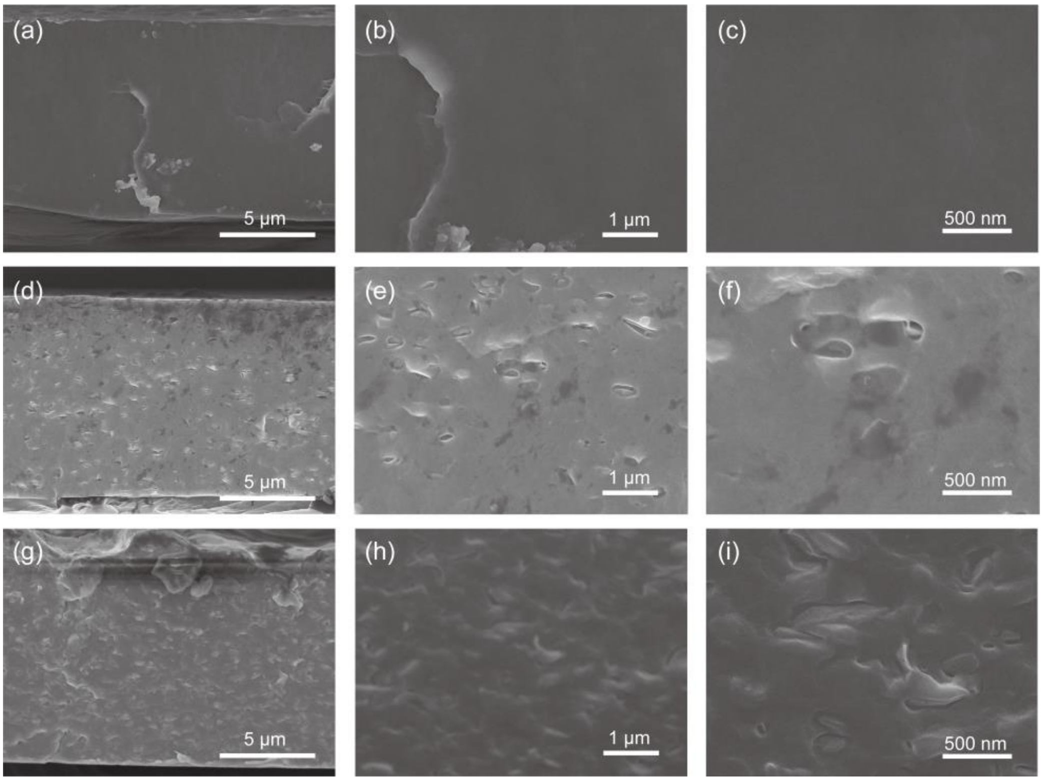

Figure S2. SEM cross section of (a-c) PVDF, (d-f) 1 wt.% and (g-h) 2 wt.% CNO/PVDF films printed under shear mode.

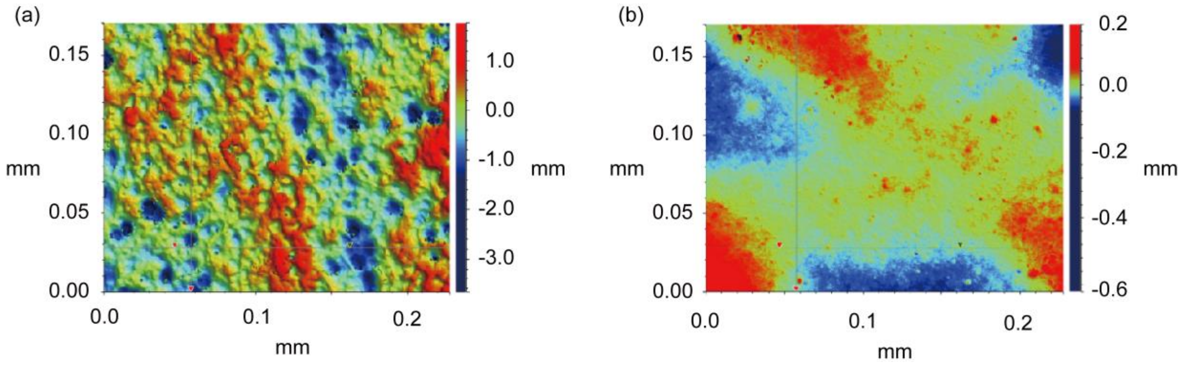

Figure S3. Surface morphology characterization of (a) PVDF and (b) 2 wt.% CNO/PVDF composite films: white light interferometry.

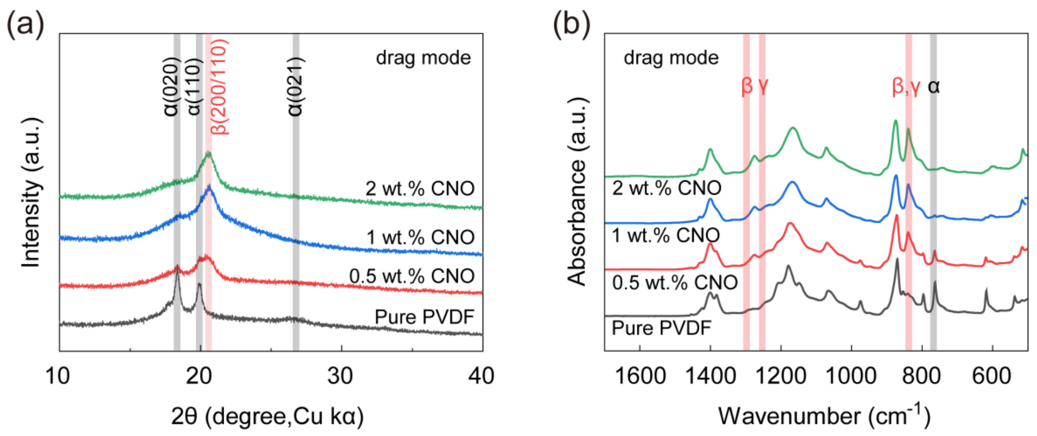

Figure S4. Structural characterization of unoriented films fabricated by drag mode: (a) XRD and (b) FTIR Spectroscopy Spectra.

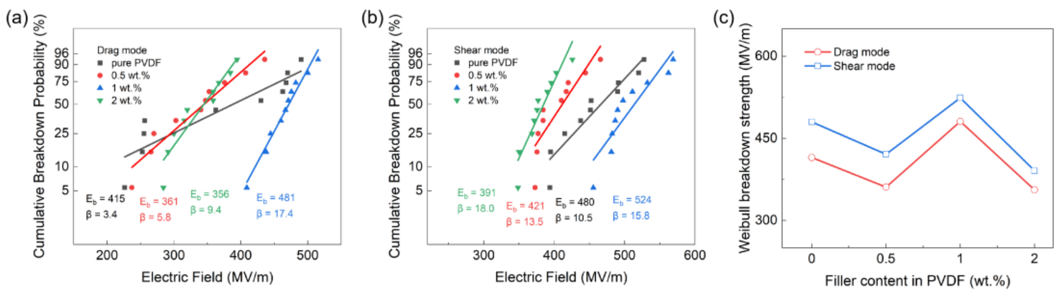

Figure S5. Comparative Analysis of Weibull Breakdown Strength for Oriented versus Unoriented Films.

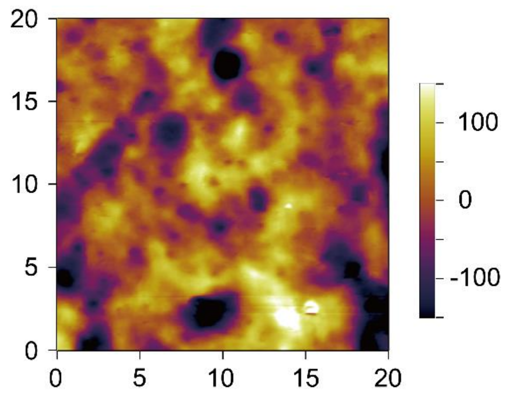

Figure S6. The AFM image of the 2 wt.% CNO-PVDF film. The scanning area is 20 μm × 20 μm.

Table S1. Enthalpy (ΔH_m), Crystallinity (χ_c), Melting Point (T_m) and Curie temperature (T_c) of DIW PVDF and PVDF - 0.5 wt.%, 1 wt.% and 2 wt.% CNO films under shear mode.

	ΔH_m (J/g)	χ_c (%)	T_m (°C)	T_c (°C)
PVDF	50.384	48.12	166.58	--
PVDF-0.5 wt.% CNO	47.865	45.72	164.07	173.78
PVDF-1 wt.% CNO	46.079	44.01	162.62	172.43
PVDF-2 wt.% CNO	48.996	46.77	161.72	170.12
